# A Blood-Based Interferon Viral Score Defines Acute RSV Bronchiolitis in Infants

**DOI:** 10.3390/idr18020029

**Published:** 2026-04-01

**Authors:** Ilaria Galliano, Stefania Alfonsina Liguori, Anna Pau, Paola Montanari, Cristina Calvi, Anna Clemente, Anna Massobrio, Claudia Linari, Stefano Gambarino, Alessandra Conio, Massimiliano Bergallo

**Affiliations:** 1 Department of Public Health and Pediatric Sciences, University of Turin, 10100 Turin, Italy; pauanna1@gmail.com (A.P.); paola.montanari@unito.it (P.M.); cristina.calvi@unito.it (C.C.); anna.clemente@unito.it (A.C.); stefano.gambarino@unito.it (S.G.); massimiliano.bergallo@unito.it (M.B.); 2 Laboratory of Specialistic Pediatry, Department of Children’s Pathology and Care, Regina Margherita Children’s Hospital, Piazza Polonia 94, 10126 Turin, Italy; 3 Early Infancy Special Care Unit, Regina Margherita Children Hospital, 10126 Turin, Italy; sliguori@cittadellasalute.to.it (S.A.L.); aconio@cittadellasalute.to.it (A.C.); 4 Laboratory Medicine, Regina Margherita Children’s Hospital, 10126 Turin, Italy; amassobrio@cittadellasalute.to.it (A.M.); clinari@cittadellasalute.to.it (C.L.)

**Keywords:** RSV bronchiolitis, interferon-stimulated genes, viral score, host-response biomarkers, pediatric respiratory infections

## Abstract

Background: Respiratory syncytial virus (RSV) is the leading cause of bronchiolitis and hospitalization in infancy. Reliable biomarkers reflecting host antiviral responses and disease dynamics are still lacking. Methods: We evaluated the expression of the interferon-stimulated genes IFI44L, IFI27, and RSAD2 in peripheral blood of infants hospitalized with RSV bronchiolitis at admission and discharge, and in healthy controls, using multiplex RT-qPCR. A composite interferon-based Viral Score was derived from coordinated ISG expression. Results: All three ISGs and the Viral Score were markedly elevated during acute RSV infection at hospital admission compared with discharge and healthy controls. Following clinical recovery, ISG expression and Viral Score declined significantly and approached baseline levels. The Viral Score clearly discriminated acute infection from recovery and healthy states, reflecting dynamic systemic interferon activation. Conclusions: A Viral Score based on IFI44L, IFI27, and RSAD2 captures systemic antiviral immune responses in infants with RSV bronchiolitis and declines with disease resolution. This interferon-based host-response signature represents a promising biomarker for defining viral infection status and monitoring disease dynamics in pediatric respiratory infections.

## 1. Introduction

Bronchiolitis is the most common acute lower respiratory tract infection in children under 24 months of age and represents the leading cause of hospitalization in infancy, particularly during the winter season [1]. Respiratory syncytial virus (RSV) accounts for the majority of bronchiolitis cases worldwide, although other respiratory viruses may contribute to disease burden. Clinical manifestations range from mild upper respiratory symptoms to severe respiratory failure requiring intensive care, reflecting substantial inter-individual variability in host immune responses and disease severity. Despite its high prevalence and clinical impact, bronchiolitis remains largely a clinical diagnosis, and reliable molecular biomarkers reflecting disease activity and underlying host antiviral responses are still lacking.

RSV is transmitted through respiratory droplets and direct contact with infected secretions, with an incubation period of approximately 4–6 days. Infection-induced immunity is incomplete and short-lived, allowing recurrent infections throughout life [2]. The pathogenesis of RSV bronchiolitis involves infection of the airway epithelium, dysregulated innate immune activation, mucus hypersecretion, and airway obstruction, leading to impaired gas exchange and respiratory distress [3]. Current management is primarily supportive, including oxygen supplementation and fluid therapy, as pharmacological interventions have shown limited benefit [4,5]. Importantly, severe RSV infection in early life has been associated with an increased risk of long-term respiratory morbidity, including recurrent wheezing and asthma [6,7], highlighting the need for biomarkers that capture disease biology and host immune responses.

Interferons (IFNs) are central mediators of antiviral immunity and play a pivotal role in shaping host responses to viral infections. IFNs are classified into three main types: type I IFNs (including IFN-α and IFN-β), type II IFN (IFN-γ), and type III IFNs (IFN-λ family) [8,9]. Type I IFNs induce a potent systemic antiviral state, whereas type III IFNs primarily act at epithelial barriers, particularly at mucosal surfaces of the respiratory tract [10]. Following viral recognition by pattern recognition receptors, IFN signaling activates the JAK–STAT pathway, leading to transcription of hundreds of interferon-stimulated genes (ISGs) that collectively inhibit viral replication and modulate innate and adaptive immune responses [11]. In particular, activation of STAT1 and STAT3 plays a key role in mediating type I interferon signaling. These transcription factors regulate the expression of multiple interferon-stimulated genes and contribute to the amplification and modulation of the antiviral immune response [11].

In RSV infection, interferon signaling plays a critical but complex role. While timely IFN induction is essential for viral control, RSV has evolved multiple mechanisms to antagonize IFN production and signaling, including the activity of non-structural proteins NS1 and NS2 [12] and the induction of suppressor of cytokine signaling (SOCS) proteins [13]. Consequently, the magnitude and quality of the interferon response may critically influence disease severity and clinical outcome. Several transcriptomic studies have shown that RSV induces a strong interferon-driven gene expression signature in blood and respiratory samples of infected infants, underscoring the systemic nature of the antiviral response [14,15].

Among ISGs, IFI27, IFI44L, and RSAD2 have emerged as particularly robust and reproducible markers of viral infection. IFI27 is one of the most strongly inducible ISGs in response to type I interferon signaling and consistently exhibits large fold changes during acute viral respiratory infections [16,17]. IFI44L has been extensively validated as a core component of minimal host-response signatures capable of discriminating viral from bacterial infections across different age groups and clinical settings [18,19]. RSAD2 (viperin) encodes a multifunctional antiviral effector protein that directly inhibits replication of several RNA viruses, including RSV, and modulates innate immune signaling pathways [20].

The coordinated regulation of these genes during acute viral infection provides the biological basis for their integration into a composite interferon-based viral score. Rather than relying on single-gene measurements, which may be affected by biological variability or technical noise, a composite score captures the magnitude of interferon pathway activation in a more robust and biologically meaningful manner. Minimal ISG-based signatures incorporating IFI27- and IFI44L-related pathways have been shown to outperform conventional inflammatory markers in defining viral infection and discriminating viral from bacterial etiologies [18,19]. The inclusion of RSAD2 further strengthens the biological relevance of the score by incorporating a gene with direct antiviral effector function.

On this basis, we defined a Viral Score derived from the combined expression of IFI27, IFI44L, and RSAD2 as an integrated measure of systemic antiviral immune activation. This score is designed to reflect active viral infection rather than residual viral detection and to dynamically track changes in host interferon responses over the course of disease.

The aim of the present study was to evaluate interferon-stimulated gene expression and the derived Viral Score in peripheral blood of infants hospitalized with RSV bronchiolitis, comparing acute disease at hospital admission, clinical recovery at discharge, and healthy controls. We hypothesized that the viral score would be markedly elevated during acute RSV infection and decline with disease resolution, supporting its use as a biologically grounded and translational biomarker of viral infection status and disease dynamics in pediatric RSV bronchiolitis.

## 2. Materials and Methods

### 2.1. Study Population

A total of 119 infants with laboratory-confirmed RSV infection were enrolled at the Neonatal and Infant Pathology Unit of the Regina Margherita Children’s Hospital in Turin during the RSV epidemic season (December 2023 to April 2024). RSV infection was confirmed using a rapid antigen detection test performed on nasopharyngeal swabs as part of routine clinical diagnostics in the hospital laboratory.

Inclusion criteria for the RSV group were: (a) age < 12 months, (b) hospitalization for clinically diagnosed bronchiolitis, and (c) laboratory-confirmed RSV infection.

Exclusion criteria included: (a) known primary or secondary immunodeficiency, (b) major congenital malformations, (c) chronic inflammatory or autoimmune diseases, and (d) insufficient blood sample for molecular analysis.

Severe disease was defined as the need for admission to the intensive care unit according to the 2022 Italian Guidelines for bronchiolitis management [4].

Peripheral blood samples were collected at hospital admission (or on the following morning in cases of late admission) and processed for analysis. Discharge samples were collected at the time of clinical recovery, defined as either 24 h after discontinuation of ventilatory support or, in patients not requiring respiratory support, at hospital discharge. In all cases, discharge sampling was performed under stable clinical conditions. Both admission and discharge samples were obtained from residual material of routine clinical blood tests, and no additional blood sampling was performed for research purposes. The healthy control group (HC) consisted of 32 age-matched, uninfected infants (14 males and 18 females; mean age 58.0 days). Healthy controls were recruited at the same hospital during routine clinical evaluations or minor elective procedures. Blood samples were obtained from residual material of routine laboratory tests. All controls were screened to exclude current or recent infections based on clinical assessment and routine laboratory parameters. No significant differences in age or sex distribution were observed between RSV-infected patients and controls (*p* = 0.8350). Exclusion criteria for the control group included suspected or confirmed infections, malignancies, autoimmune diseases, neurological disorders, or abnormal laboratory results.

The study was designed to evaluate (a) within-subject changes in interferon-stimulated gene expression between the acute phase (hospital admission) and clinical recovery (discharge), and (b) the ability of the Viral Score to discriminate RSV infection from healthy controls. The potential clinical application of this score as a dynamic biomarker of infection status and disease monitoring was also explored.

### 2.2. Sample Storage

Within one hour from collection, samples were stored in RNA-stabilizing solution as following: 200 μL of whole blood were added to 800 µL of RNApro solution (Biomole, Turin, Italy) in a 1.5 mL tube [21]; samples were vortexed and stored at −80 °C until extraction.

### 2.3. Total RNA Extraction

Total RNA was extracted from whole blood using the Maxwell automated extractor (Promega, Madison, WI, USA) with the RNA Blood Kit (Promega, Madison, WI, USA), which includes DNase treatment.

In each extraction session, a negative control was set up with sterile water, as potential contamination control. RNA concentration and purity were assessed via UV spectrophotometry at 260/280 nm, using the Beer-Lambert law for quantification. NanoDrop (Thermo Fisher Scientific, Waltham, MA, USA) confirmed RNA concentrations within the acceptable range. Measurements were taken with 1 μL of RNA on an ND-1000 spectrophotometer (Thermo Fisher Scientific, Waltham, MA, USA) at room temperature, with an A260/A280 ratio of 1.8–2.1 indicating high purity. Although spectrophotometry evaluates RNA purity rather than structural integrity, sample quality was indirectly supported by consistent Ct values of the housekeeping gene GAPDH across samples and by strict adherence to standardized storage and handling procedures. Formal RNA integrity assessment (e.g., agarose gel electrophoresis or RIN analysis) was not performed. RNA extracts were amplified without reverse transcription for GAPDH, under the same thermal cycling conditions applied for RT-qPCR (40 cycles), to rule out genomic DNA contamination. The absence of amplification confirmed that no significant genomic DNA contamination was present. A no-template control was included in each PCR run and consistently showed no signal. RNA extracts were stored at −80 °C until use and were analyzed within two weeks from extraction. For all procedures we followed the best practices for RNA storage and sample handling.

### 2.4. Reverse Transcription

Four hundred nanograms of total RNA were reverse-transcribed with 2 μL of buffer 10×, 4.8 μL of MgCl2 25 mM, 2 μL ImpromII (Promega), 1 μL of RNase inhibitor 20 U/L, 0.4 μL random hexamers 250 μM (Promega), 2 μL mix dNTPs 100 mM (Promega), and dd-water in a final volume of 20 μL. The reaction mix was carried out in a GeneAmp polymerase chain reaction (PCR) system 9700 Thermal Cycle (Thermo Fisher Scientific, Waltham, MA, USA) under the following conditions: 5 min at 25 °C, 60 min at 42 °C and 15 min at 70 °C for the inactivation of enzyme; the cDNAs were stored at −80° until use.

### 2.5. Transcription Levels of Virus Score by RT-PCR

GAPDH was chosen as the reference gene due to its stability and previous use in our studies [22,23]. Relative quantification of mRNA for ISGs: IFI27, IFI44L, and RSAD2 were performed using the ABI PRISM 7500 real-time system (Thermo Fisher Scientific, Waltham, MA, USA).

40 ng of cDNA were quantified by real-time PCR in a 20 μL of total volume reaction containing 2.5 U goTaQ MaterMix (Promega), 1.25 mmol/L MgCl2, 500 nmol of specific primers and 200 nmol of specific probes. The IFI27 primers were: (IFI27F 5′-TGTCATTGCGAGGTTCTACTAGCT-3′) (IFI27R 5′-CCCCTGGCATGGTTCTCTT-3′), and the probe was: (IFI27P TEXAS-RED-CCTGCCCCTCGCCCTGCA-BHQ2). The IFI44L primers were: (IFI44LF 5′-TGCAGTGAGGTTCTTCAAGACAA-3′) (IFI44LR 5′-CATCAAAATATTGGAAATAGGAATGC-3′), and the probe was: (IFI44LP TEXAS-RED-CTCAAAGCCGGGTCATGAATGTCCA–BHQ2). The RSAD2 primers were: (RSAD2F 5′-GAGGGCCAGATGAGACCAAA-3′) (RSAD2R 5′-GTGAAGTGATAGTTGACGCTGGTT-3′), and the probe was: (RSAD2P CY3-AGGACCCTCCTCTGCCCACCACC-BHQ2). The GAPDH primers were: (GAPDHF 5′–ATGCTGGCGCTGAGTACGT–3′) (GAPDHR 5′–AGCCCCAGCCTTCTCCAT–3′), and the probe was: (GAPDHP CY5-TGGAGAAGGCTGGGGCTCAT-BHQ2). The primers and probes were designed by Primer ExpressTM software version 3.0 (Applied Biosystems, Foster City, CA, USA).

Reactions were performed in a 96-well plate with the following thermal profile: 50 °C for 10 min, 95 °C for 10 min, followed by 40 cycles of 95 °C for 10 s and 60 °C for 30 s.

The integrity and adequacy of RNA extracts was evaluated by analyzing the Cycle threshold (Ct) obtained in the PCR for the Housekeeping gene GAPDH in all samples. Each sample was analyzed in technical triplicate to ensure reproducibility. Ct values obtained for GAPDH and target genes were consistent across replicates and fell within a repeatable range, supporting the quality of RNA extracts and amplification reliability. Ct values of the housekeeping gene GAPDH showed minimal variability across samples and did not differ substantially between clinical groups, supporting the stability of GAPDH as reference gene in this cohort. No samples showed undetectable amplification for the analyzed targets, and therefore no reactions were excluded from quantitative analysis. Primer and probe sets were designed using Primer Express software and previously validated in our laboratory to ensure specificity and efficient amplification.

Relative gene expression was calculated using the ΔΔCt method [24], and results were expressed as relative quantification (RQ) values in arbitrary units. Briefly, Ct values of each target gene were first normalized to the housekeeping gene GAPDH (ΔCt). ΔΔCt values were then obtained by calibrating each sample against the median expression of the corresponding gene in the healthy control group. RQ values were calculated as 2^−ΔΔCt^, representing fold changes relative to controls.

The Viral Score was defined as the median of the normalized relative expression (RQ) values of the selected interferon-stimulated genes (ISGs: IFI44L, IFI27 and RSAD2) for each individual sample. This composite score provides an overall measure of interferon pathway activation, serving as an indirect marker of the host antiviral transcriptional activity.

All analyses were conducted in a Biosafety Level 2 (BSL-2) laboratory, following NIH and WHO guidelines (NIH, 2024; WHO-WPE-GIH, 2021) [25,26].

### 2.6. Statistical Analysis

Data distribution was assessed using the Shapiro–Wilk test. As most variables did not follow a normal distribution, results are presented as median and interquartile range (IQR), and non-parametric tests were applied.

Comparison of Viral Scores among admission, discharge, and control samples were performed using the Kruskal–Wallis test followed by Dunn’s multiple comparisons test for multiple testing. Within-subject comparisons between admission and discharge samples were analyzed using the Wilcoxon matched-pairs signed-rank test. Differences between severe and mild cases were analyzed using the Mann–Whitney U test. Categorical clinical variables, including prematurity, previous NICU admission, and co-infections, were compared using Fisher’s exact test. Effect size for the Mann–Whitney comparison was calculated as rank-biserial correlation (r_rb_ = 1 − 2U/(n_1_ × n_2_)).

All tests were two-tailed. Statistical analyses were performed using GraphPad Prism version 7 (GraphPad Software, Boston, MA, USA), and *p* value < 0.05 was considered statistically significant.

## 3. Results

### 3.1. Study Population

The cohort included 58 males and 61 females, with no significant sex-related differences, and a mean age of 65.0 days. Among the enrolled patients, 18 were born preterm, and 26 had a previous history of admission to a neonatal intensive care unit.

Regarding respiratory support, 78 infants required high-flow nasal cannula (HFNC), two received continuous positive airway pressure (C-PAP), and one was treated with bubble C-PAP. The majority of patients (100 infants, 84.0%) were receiving inhaled corticosteroid therapy, according to local clinical practice, mainly in the presence of wheezing or suspected bronchial hyperreactivity. These treatments were administered by inhalation and not systemically, and no patient received systemic corticosteroids. A total of 18 patients (15.1%) required admission to the intensive care unit (ICU) and were therefore classified as having severe disease, while the remaining 101 patients were classified as mild cases.

The median duration of hospitalization was 8 days (IQR 6–10), and the median time between admission and discharge sampling was 5 days (IQR 3.8–7), reflecting the expected clinical variability in recovery time among patients.

The main demographic and clinical characteristics of the cohort are summarized in Table 1.

Potential clinical confounders were explored descriptively between mild and severe cases. No significant differences were observed for co-infections (*p* = 0.1359), prematurity (*p* = 0.7351), previous NICU admission (*p* = 0.5401), or time since symptom onset at admission (*p* = 0.2566).

Viral co-infections had been previously evaluated in this cohort using nasopharyngeal swabs tested for common respiratory viruses, as described in our previous study [27]. Briefly, 10 patients (8.4%) presented viral co-infections, including coronaviruses, rhinovirus, influenza A, and SARS-CoV-2. Given the low proportion of co-infected patients, separate statistical analyses were not considered meaningful and subsequent analyses were performed on the entire bronchiolitis cohort.

### 3.2. Interferon-Stimulated Gene Expression in Infants with RSV Bronchiolitis

**Figure 1:**
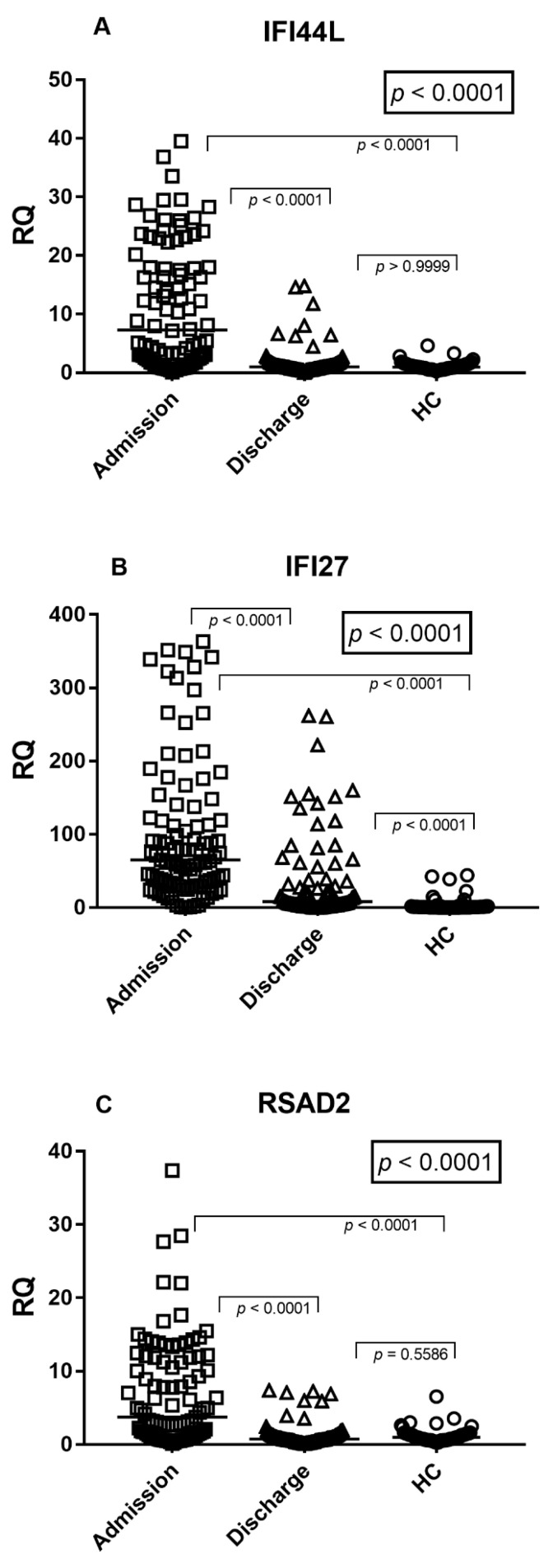
Interferon-stimulated gene expression in infants with RSV bronchiolitis. (**A**) IFI44L; (**B**) IFI27; (**C**) RSAD2 expression in peripheral blood samples from infants with RSV bronchiolitis at hospital admission and hospital discharge, compared with healthy controls (HC). Each dot represents an individual subject; horizontal lines indicate median values. Statistical analysis was performed using the Kruskal–Wallis test followed by Dunn’s multiple comparisons test.

The expression levels of the interferon-stimulated genes IFI27, IFI44L, and RSAD2 were evaluated in peripheral blood samples from infants hospitalized with RSV bronchiolitis at hospital admission and at hospital discharge and compared with healthy controls (HC) (Figure 1A–C).

Overall group comparisons were performed using the Kruskal–Wallis test followed by Dunn’s multiple comparisons test. In addition, within-subject changes between admission and discharge were assessed using the Wilcoxon matched-pairs signed-rank test.

At hospital admission, IFI44L expression was significantly higher than both discharge samples and healthy controls (Dunn’s test *p* < 0.0001 for both comparisons; Figure 1A). In paired analyses, IFI44L expression significantly decreased from admission to discharge (Wilcoxon test, *p* < 0.0001) and did not differ from healthy controls at discharge (*p* > 0.9999).

IFI27 expression showed a similar increase during the acute phase, with admission levels significantly higher than both discharge and healthy controls (Dunn’s test *p* < 0.0001 for both comparisons; Figure 1B). In paired analysis, IFI27 transcript levels decreased at hospital discharge (Wilcoxon test, *p* < 0.0001 vs. admission), although they remained significantly higher than those observed in healthy controls (Dunn’s test, adjusted *p* < 0.0001).

Consistent with IFI44L, RSAD2 expression was significantly elevated at hospital admission compared with discharge samples and healthy controls (Dunn’s test, *p* < 0.0001 for both comparisons; Figure 1C). RSAD2 levels significantly declined at discharge (Wilcoxon test, *p* < 0.0001) and were not significantly different from healthy controls (*p* = 0.5586).

Overall, IFI44L and RSAD2 displayed a marked normalization by discharge, whereas IFI27 remained persistently elevated compared with healthy controls, despite a significant decrease from admission.

The median values and the interquartile ranges (median, IQR 25–75%) were: IFI44L: Admission = 7.69, 2.08–18.57; Discharge = 1.04, 0.73–1.58; HC = 0.99, 0.73–1.42. IFI27: Admission = 67.12, 33.08–138.30; Discharge = 8.56, 2.87–37.60; HC = 0.89, 0.46–1.72. RSAD2: Admission = 3.96, 1.30–11.84; Discharge = 0.76, 0.52–1.29; HC = 0.97, 0.62–1.42.

### 3.3. Definition and Distribution of the Interferon-Based Viral Score (Revised)

To integrate the coordinated expression of IFI44L, IFI27, and RSAD2, a composite interferon-based Viral Score was calculated for each sample, as described in the Materials and Methods section. The Viral Score represents the median of the normalized relative expression (RQ) values of the three interferon-stimulated genes (IFI44L, IFI27, and RSAD2), as calculated using the 2^−ΔΔCt^ method and described in the Materials and Methods section.

**Figure 2:**
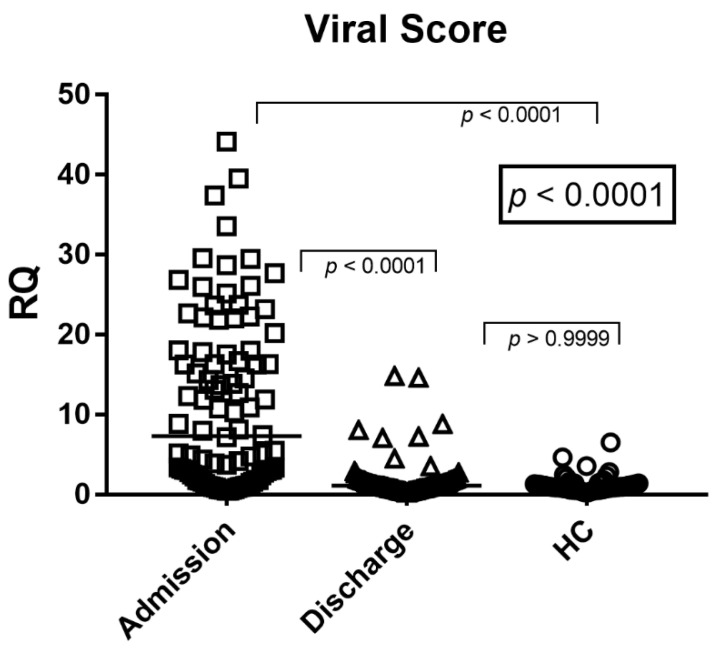
Viral Score expression in infants with RSV bronchiolitis.

As shown in Figure 2, the Viral Score was significantly higher in infants with RSV bronchiolitis at hospital admission compared with both discharge samples and healthy controls (Dunn’s test *p* < 0.0001 for both comparisons). This finding reflects the strong activation of the interferon response during the acute phase of RSV infection.

In paired analysis, the Viral Score was significantly reduced compared with admission values (Wilcoxon test *p* < 0.0001; Figure 2). Viral Score values at discharge were not significantly different from those observed in healthy controls (Dunn’s test *p* > 0.05), indicating a substantial normalization of systemic interferon activity following clinical recovery. The effect size for the primary comparison (Viral Score at admission vs. healthy controls) was large (r_rb_ = 0.64).

Overall, the Viral Score was significantly elevated during acute RSV infection at admission and decreased to levels comparable to healthy controls at hospital discharge.

The median values and the interquartile ranges (median, IQR 25–75%) of the Viral Score were: Admission = 7.69, 2.14–17.98; Discharge = 1.11, 0.74–1.71; HC = 0.99, 0.70–1.38.

The interferon-based Viral Score, calculated as the median expression of IFI44L, IFI27, and RSAD2, is shown in peripheral blood samples from infants with RSV bronchiolitis at hospital admission and hospital discharge, compared with healthy controls (HC). Each dot represents an individual subject; horizontal lines indicate median values. Statistical analysis was performed using the Kruskal–Wallis test followed by Dunn’s multiple comparisons test.

### 3.4. Interferon-Stimulated Gene Expression and Viral Score According to Disease Severity

To evaluate the relationship between interferon response and disease severity, expression levels of IFI44L, IFI27, RSAD2, and the composite Viral Score were compared between infants with mild disease and those requiring intensive care (severe cases).

**Figure 3:**
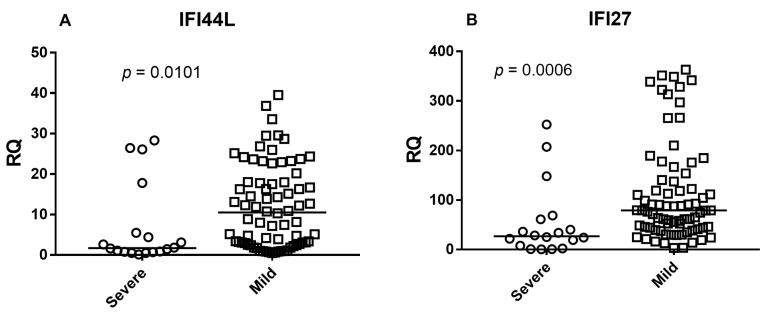

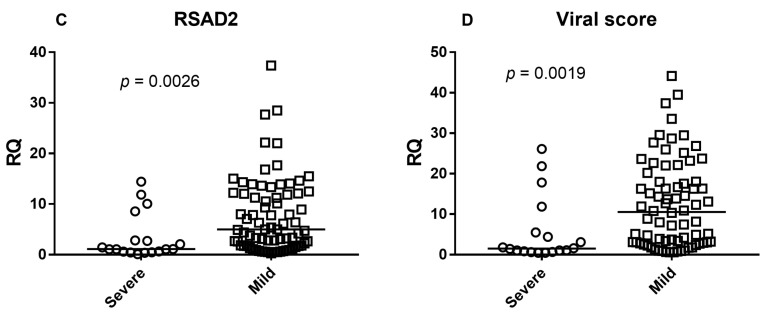
Interferon-stimulated gene expression and Viral Score according to disease severity. Expression levels of (**A**) IFI44L, (**B**) IFI27, (**C**) RSAD2, and (**D**) the composite Viral Score in infants with RSV bronchiolitis stratified by disease severity (mild vs. severe/ICU). Each dot represents an individual subject and horizontal lines indicate median values. Statistical comparisons were performed using the Mann–Whitney U test.

Expression levels of all three interferon-stimulated genes were significantly higher in mild cases compared with severe cases (Mann–Whitney U test: IFI44L *p* = 0.0101; IFI27 *p* = 0.0006; RSAD2 *p* = 0.0026) (Figure 3A–C).

Consistently, the Viral Score was also significantly higher in mild cases than in severe cases (*p* = 0.0019) (Figure 3D).

These findings indicate that infants with milder disease exhibited higher systemic interferon-stimulated gene expression and higher Viral Scores at hospital admission.

## 4. Discussion

In this study, we demonstrate that infants hospitalized with RSV bronchiolitis exhibit a pronounced systemic type I interferon response at the time of hospital admission, as reflected by markedly elevated expression of the interferon-stimulated genes IFI27, IFI44L, and RSAD2 in peripheral blood. As shown in Figure 1A–C, all three ISGs were significantly upregulated during the acute phase of disease compared with samples obtained at hospital discharge and from healthy controls. Consistently, the composite viral score derived from these genes was markedly elevated at admission and declined significantly at discharge, approaching baseline levels observed in healthy controls (Figure 2). Notably, while IFI44L and RSAD2 expression largely normalized by hospital discharge, IFI27 levels remained significantly higher than in healthy controls. This finding likely reflects the higher sensitivity and slower normalization kinetics of IFI27 in response to interferon signaling, as previously described, and may represent ongoing resolution of interferon activity rather than active viral replication [16,17]. Taken together, these findings indicate that interferon-driven transcriptional activity closely mirrors disease dynamics in RSV bronchiolitis and can be captured through a concise, biologically grounded viral score.

The interferon signature observed in our cohort is consistent with previous transcriptomic studies reporting robust activation of interferon pathways in infants with RSV infection [14,15]. Although RSV primarily infects the respiratory epithelium, systemic immune activation is a well-recognized feature of clinically significant disease, and blood-based ISG signatures effectively reflect this response. Importantly, the clear reduction in both individual ISG expression and viral score at hospital discharge supports the concept that interferon signaling diminishes as viral replication is controlled and inflammation resolves. This temporal pattern reinforces the interpretation of the viral score as a marker of active viral infection rather than residual viral nucleic acid detection.

A central strength of this work lies in the definition and application of a composite viral score based on IFI27, IFI44L, and RSAD2. Rather than relying on single-gene measurements, which may be influenced by inter-individual variability or technical fluctuations, the viral score integrates the coordinated expression of multiple interferon-responsive genes. As demonstrated in Figure 1A–C, these genes displayed highly concordant expression patterns across clinical conditions, indicating that they are co-regulated components of the interferon pathway. Their integration into a single score therefore provides a more stable and biologically meaningful readout of systemic antiviral immune activation.

Each gene included in the viral score contributes distinct and complementary information. IFI27 is among the most strongly inducible ISGs during acute viral respiratory infections and exhibits a wide dynamic range in whole blood, making it particularly sensitive to changes in interferon signaling [16,17]. IFI44L has emerged as a core component of validated host-response signatures discriminating viral from bacterial infections across diverse cohorts and age groups [18,19]. Beyond its diagnostic value, IFI44L has been shown to restrict RSV replication, supporting its functional relevance in antiviral defense [19]. RSAD2 encodes viperin, a multifunctional antiviral effector protein that directly inhibits viral replication and modulates innate immune signaling pathways, adding biological depth to the composite score [20]. The combination of these genes therefore captures both the magnitude and functional relevance of the interferon-mediated antiviral response.

The clear separation observed between admission, discharge, and healthy control samples for the viral score (Figure 2) underscores its potential utility as a quantitative and dynamic biomarker. The high values observed at admission reflect intense systemic interferon activation during acute RSV infection, while the marked decline at discharge parallels clinical recovery and resolution of inflammation. This dynamic behavior is particularly relevant in pediatric RSV bronchiolitis, where viral RNA may persist for prolonged periods after symptom resolution [28]. In this context, host-response biomarkers such as the viral score may provide clinically meaningful information beyond pathogen detection alone, reflecting ongoing immune engagement and disease activity.

From a translational perspective, the viral score addresses several limitations of current diagnostic approaches. Pathogen-directed PCR assays identify viral nucleic acids but do not discriminate between active infection and residual viral shedding, nor do they provide information on host response or disease trajectory. In contrast, an interferon-based viral score integrates host biology and captures the systemic antiviral state. Previous studies have shown that minimal ISG-based signatures outperform conventional inflammatory markers in defining viral infections and distinguishing viral from bacterial etiologies [18,19]. Our findings extend this concept to RSV bronchiolitis, demonstrating that a small, targeted ISG panel can effectively define acute viral infection and monitor disease resolution.

Notably, this observation is consistent with our previous study in a similar RSV-positive pediatric cohort, in which severe cases showed significantly lower type I interferon signature levels in both blood and nasal samples [27]. However, the relatively small number of severe patients warrants cautious interpretation, and larger prospective studies will be needed to better define the prognostic value of interferon-based biomarkers in RSV bronchiolitis.

An additional aspect of interest is the relationship between interferon responses, viral score magnitude, and disease severity. Several studies have reported that impaired or delayed type I interferon responses are associated with severe RSV disease [29], as well as with severe influenza and COVID-19 [30,31]. In line with these observations, in our cohort mild cases exhibited significantly higher expression levels of IFI44L, IFI27, RSAD2, as well as higher Viral Scores compared with severe cases requiring ICU admission. This finding suggests that a more robust systemic interferon response may be associated with more effective viral control and a milder clinical course. Notably, this observation is consistent with our previous study in a similar RSV-positive pediatric cohort, in which severe cases showed significantly lower type I interferon signature levels in both blood and nasal samples [27]. However, the relatively small number of severe patients warrants cautious interpretation, and larger prospective studies will be needed to better define the prognostic value of interferon-based biomarkers in RSV bronchiolitis.

The use of a composite viral score may also facilitate standardization across platforms and clinical settings. By integrating multiple ISGs with complementary characteristics, the score is less sensitive to technical variability affecting individual targets and more robust to biological heterogeneity across patients. This feature is particularly important in pediatric populations, where age-related immune maturation and differences in baseline interferon activity may influence single-gene expression levels. A composite score therefore provides a more reliable framework for translating host-response biomarkers into clinical practice.

Given its limited gene set and robust dynamic behavior, this interferon-based viral score may be amenable to future translation into rapid, host-response–based point-of-care assays, pending further validation.

This study has limitations that should be acknowledged. A subset of the patients included in the present study was part of a previously published cohort in which broader type I and III interferon signatures were analyzed; however, the present work addresses a distinct research objective by focusing on a simplified three-gene Viral Score with potential translational applicability as a dynamic biomarker of systemic antiviral activation. The cohort size, particularly for severe cases, limits subgroup analyses, and the exclusive focus on transcriptomic data precludes direct assessment of interferon protein levels or functional antiviral activity. In addition, a high proportion of patients received inhaled corticosteroid therapy according to local clinical practice. Although corticosteroids may influence interferon signaling pathways, most evidence refers to systemic administration, whereas inhaled corticosteroids are expected to have limited systemic immunomodulatory effects. Finally, the present study focused exclusively on RSV infection; inclusion of other viral and bacterial respiratory infections will be required to formally validate the specificity and discriminatory performance of the viral score in broader clinical contexts.

## 5. Conclusions

In conclusion, our data demonstrate that interferon-stimulated gene expression in blood, specifically IFI27, IFI44L, and RSAD2, is markedly elevated during acute RSV bronchiolitis in infants and declines with clinical recovery. The definition of a composite viral score based on these genes provides a robust, biologically grounded measure of systemic antiviral immune activation. This interferon-based host-response signature represents a promising biomarker for defining viral infection status, monitoring disease dynamics, and supporting the development of host-response–guided diagnostic and management strategies in pediatric RSV bronchiolitis.

## Figures and Tables

**Table 1:** Demographics and clinical characteristics of patients with Bronchiolitis and healthy controls.

Characteristic	Bronchiolitis (*n* = 119)	Healthy Controls (HC) (*n* = 32)
Sex (*n*, %)		
Male	58 (48.7)	14 (43.8)
Female	61 (51.3)	18 (56.2)
Age, mean ± SD (days)	65.0 ± 40.2	58.0 ± 36.2
Severe (ICU), *n* (%)	18 (15.1)	
Mild (non-ICU), *n* (%)	101 (84.9)	
Preterm infants, *n* (%)	18 (15.1)	
Time since symptom onset, mean ± SD (days)	3.2 ± 1.3	
History of neonatal ICU admission, *n* (%)	26 (21.8)	
Respiratory support, n (%)		
HFNC	78 (65.5)	
C-PAP	2 (1.6)	
Bubble C-PAP	1 (0.8)	
Inhaled steroid treatment, *n* (%)	101 (84.9)	
Co-infections, *n* (%)	10 (8.4)	

*n*: number; SD: standard deviation; ICU: intensive care unit; HFNC: high flow nasal cannula; C-PAP: continuous positive airway pressure.

## Data Availability

The data presented in this study are available from the corresponding author upon reasonable request, due to ethical and privacy restrictions.
